# Water Resistance of Compressed Earth Blocks Stabilised with Thermoactivated Recycled Cement

**DOI:** 10.3390/ma17225617

**Published:** 2024-11-17

**Authors:** Ricardo Cruz, José Alexandre Bogas, Andrea Balboa, Paulina Faria

**Affiliations:** 1 CERIS—Civil Engineering Research and Innovation for Sustainability, Instituto Superior Técnico, Universidade de Lisboa, Av. Rovisco Pais, 1, 1049-001 Lisboa, Portugal; jose.bogas@tecnico.ulisboa.pt (J.A.B.); andrea.ferre.balboa@tecnico.ulisboa.pt (A.B.); 2 CERIS—Civil Engineering Research and Innovation for Sustainability, NOVA School of Science and Technology, University of Lisbon, 2829-516 Caparica, Portugal; paulina.faria@fct.unl.pt

**Keywords:** earth masonry unit, CEB, chemical stabilisation, compressive strength, recycled binder, water durability, eco-efficiency

## Abstract

Low water resistance is the main shortcoming of unfired earth materials, requiring chemical stabilisation for some durable applications. Ordinary Portland cement (PC) is an efficient stabiliser, but it goes against the ecological and sustainable nature of earth construction. This study explores the use of low-carbon thermoactivated recycled cement (RC) obtained from old cement waste as a new eco-efficient alternative to PC in the stabilisation of compressed earth blocks (CEBs). The objective is to improve the durability of the CEB masonry even when applied in direct contact with water, without compromising its eco-efficiency. The water resistance of the CEBs with 0% (unstabilised) and 5% and 10% (wt. of earth) stabiliser and partial to total replacement of PC with RC (0, 20, 50, 100% wt.) was evaluated in terms of compressive strength under different moisture contents, immersion and capillary water absorption, low-pressure water absorption, water permeability and water erosion. Low absorption and high resistance to water erosion were achieved in stabilised CEBs, regardless of the type of cement used. The incorporation of RC increased the total porosity and water absorption of the CEBs compared to PC, but significantly improved the water resistance of the unstabilised blocks. The eco-friendlier RC proved to be a promising alternative to PC stabilisation.

## 1. Introduction

Climate change and the race to net-zero carbon emissions are the main challenges the construction industry is facing nowadays. Therefore, a priority is to develop more sustainable materials and spread eco-efficient construction techniques. From this perspective, traditional earth construction has re-emerged as an affordable and eco-friendly building choice. Earth, extracted from the ground soil and often available from construction excavation works, is an abundant building material, easy to obtain, environmentally friendly and reusable and has low embodied energy. Indeed, earth stands out with a primary energy impact value over 30 times lower than that of concrete and fired bricks [1]. In addition, earthen building products can provide aesthetic, hygrothermal and other indoor environment benefits [2,3,4].

However, earth is susceptible to water, which reduces its physical, mechanical and durability properties [5,6]. To address this issue, unstabilised compressed earth blocks (UCEBs), produced with low water content and compression, emerged as a modern successor of the traditional unstabilised adobe. This technique involves mechanical processes (compression). When stabilised compressed earth blocks (SCEBs) are produced, chemical stabilisation (addition of a low content of a binder) is also included, which in general results in greater processing speed, less variability [7] and improved mechanical strength and durability compared to other earthen blocks used to build masonries [8,9]. The integrity and mechanical properties of UCEBs and SCEBs increase with compaction pressure [10]. However, only chemical stabilisation can prevent the disintegration or serious erosion of blocks exposed to water [11,12].

Although not the oldest [13], nowadays Portland cement (PC) is the most widely used inorganic binder for stabilising earth materials [14,15], especially in sandy-clay soils with up to about 20 wt% clay [16,17]. Although depending on the earth used, for usual amounts of 5–10 wt% of dry earth [6,18], PC seems to be a very effective stabiliser in the production of SCEBs, improving their volumetric stability, mechanical behaviour and durability in dry and saturated conditions [2,11,19]. For up to 10 wt% of PC, Van Damme and Houben [20] recorded a 2- to 3-fold improvement in the mechanical strength of SCEBs compared to unstabilised CEBs. In addition, Ouedrago et al. [21] reported that higher amounts of PC have a much greater effect on water resistance than on mechanical strength.

However, the use of PC in earth construction goes against the ecological and sustainable philosophy of this technique, since cement production consumes a lot of energy and natural resources and is carbon-intensive, releasing around 7% of the global anthropogenic CO_2_ emissions [22]. More eco-efficient alternative stabilisers are therefore needed to recover the low embodied energy and ecological nature of earth construction, while ensuring its good mechanical performance and durability when earthen products are applied in contact with water. Various authors have explored more sustainable stabilisers, such as organic natural tree resins [23], cow dung [24,25], or inorganic pozzolanic materials [26,27], but in general they have failed to effectively replace PC due to low availability, economic viability and performance.

Low-carbon recycled cement (RC), retrieved from the cement fraction of concrete waste, has emerged as a more environmentally friendly alternative to PC, contributing to a significant cut in CO_2_ emissions and lower consumption of natural resources, while reusing construction and demolition waste [28,29]. Basically, the binding properties of cement waste are recovered through thermoactivation at relatively low temperature, around 600–700 °C [30,31], generating α’_H_-C_2_S as the major anhydrous compound of RC [32]. According to Real et al. [33], it is estimated that the global warming potential (kg CO_2eq_) of RC production can be more than 70% lower than that of PC because the decarbonation phase is avoided and the treatment temperature is about half that of clinker manufacturing. RC is a porous material with an almost 10 times larger surface area than PC, which contributes to its high water demand and fast reactivity [29,34,35]. To date, studies on RC have been essentially limited to its incorporation into cement pastes, mortars and, to a lesser extent, concrete. The high rehydration capacity of RC has been demonstrated in cement pastes, which essentially reacts for 1–3 days, presenting mechanical strength similar to that of PC at this age [30,35,36]. However, long-term mechanical strength is generally reduced with increasing RC content, which tends to develop a smaller volume of hydration products than PC [29,34,37]. Nonetheless, it is reported that RC can be comparable to low-grade PC of strength class 32.5 [32,37,38]. Moreover, the mechanical strength of concrete is not significantly affected up to a 40% replacement of PC by RC [39]. A comprehensive review on the production and characterisation of RC is presented elsewhere [29]. Studies exploring the use of RC in the stabilisation of CEBs are still scarce. The authors’ previous work on the mechanical characterisation of CEBs with RC is noteworthy [40]. RC was found to have a high rehydration capacity, more than doubling the mechanical strength when compared to unstabilised CEBs. However, the durability of RC-added CEBs still needs to be investigated.

This study aims to analyse, for the first time, the effect of RC on the water resistance of CEBs. To this end, CEBs with different stabiliser contents (0, 5, 10 wt% of solids) and partial to total replacement of PC with RC (0, 20, 50, 100 wt% of stabiliser) were characterised in terms of immersion and capillary water absorption, low-pressure water absorption, water permeability and accelerated erosion by a water jet (spray test). Water susceptibility was also assessed through compressive strength tests on the CEBs with different water contents.

## 2. Experimental Programme

### 2.1. Earth Characterisation

Clayey sandy earth collected from a homestead near Montemor-o-Novo, Portugal, was used to produce CEBs. The earth was characterised in terms of Atterberg limits [41], dry density [42], particle size distribution [43], organic matter and optimum water content [44]. The earth was composed of 20.1% fine gravel (2 mm < d < 6 mm), 48.4% sand (75 µm < d < 2 mm) and 31.5% silt and clay (<75 µm), which is within the recommended grading range for CEB production, according to XP P 13-901 [45]. The percentage of organic matter was less than 1%, which complies with the 2% limit recommended in ARS 1333 [46] and Walker [17]. The particle dry density of the earth was 2.7 g/cm^3^ and the liquid limit (LL), plastic limit (PL) and plasticity index (PI) were 30%, 22% and 8%, respectively, which fall within the range suggested in the literature for CEBs (25–40 LL and 2–25 PI) [45,46]. The optimum water content (w_opt_), determined from the standard effort Proctor test, considering a light compaction process in a small mould [8,47], was 16% for a dry density of 1.8 g/cm^3^, which is at the upper limit suggested for CEB production [48]. The main crystalline phases were detected by X-ray diffraction (XRD) analysis, through a conventional PANalytical X’Pert Pro diffractometer with CuK_α_ radiation produced in 35 mA and 45 kV operating conditions. The samples were scanned in the 5–70° 2θ range, with a step size of 0.033° and a step time of 148 s. The main crystalline components were quartz, albite and clay minerals (muscovite and nontronite) [49].

**Figure 1:**
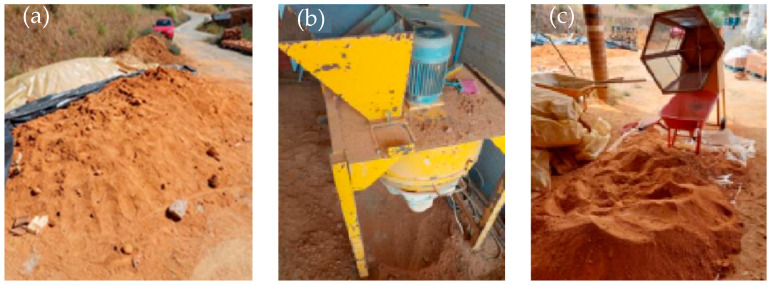
Earth preparation: (**a**) air drying; (**b**) pulverisation; (**c**) sieving.

Before CEB production, the earth was air-dried (Figure 1a), pulverised (Figure 1b) and sieved through an 8 mm mesh mechanical sieve (Figure 1c).

### 2.2. Binder Characterisation

Recycled cement (RC) was retrieved from laboratory-made pastes produced with cement type I 42.5R (PC) and a water/binder (w/b) of 0.45. It was found that RC properties are little affected by cement waste with w/b between 0.35 and 0.55 [50]. After curing for at least 120 days, the recycling process of hydrated cement paste entailed four steps: crushing, grinding, sieving and thermoactivation. The first three steps aimed to reduce the size of the cement waste particles to less than 125 μm, close to the PC fineness. The cement waste was then thermoactivated to restore its binding properties, following the procedure defined in Bogas et al. [40], which consisted of heating the material in a rotary kiln at 10 °C per minute up to 650 °C, where it remained for 3 h, followed by cooling inside the kiln to room temperature. The whole process took approximately 24 h. The main physical and chemical properties of PC and RC are summarised in Table 1. The chemical composition was determined by X-ray fluorescence (XRF) analysis. The equipment used was a Bruker XRF S4 spectrometer. The test was carried out on pre-moulded granules, apart from the lighter components analysed in pressed pellets (with mass corrected through the loss on ignition test, according to EN 196-2 [51]).

The water demand and initial setting time of RC pastes were around 2.3 and 1.7 times higher than those of PC pastes, respectively (Table 1). The setting delay is in line with the findings of other researchers [30,55] and is essentially attributed to the lower fineness and agglomeration of RC particles, as well as their phase composition [32]. In fact, RC is mainly composed of α’_H_-C_2_S, which reacts mostly after 24 h [32], unlike C_3_S in PC that reacts faster. A major shortcoming of RC is its high water demand, which is attributed to the large surface area, porous nature and hydration of the free lime (CaO) of the RC particles (Table 1) [35,36,55,56]. The mechanical strength of RC was also assessed in pastes with normal consistency (w/b = 0.73), leading to 3- and 28-day compressive strengths of 9.8 MPa and 17.3 MPa, respectively. This shows a good rehydration capacity, as found in other studies [30,37,57]. In a previous work [30], the 28-day compressive strength of a similar RC paste was around 72% of that of a PC paste of equal w/b. This was attributed to the formation of a lower volume of long-term hydration products in the RC pastes [36].

### 2.3. Compositions and Production of CEB

UCEBs and SCEBs were produced with the earth and different types (PC, RC) and amounts of stabiliser (0, 5, 10 wt% of earth). The compositions are listed in Table 2 and the SCEBs were named according to the type and amount of stabiliser: 5% and 10% PC (PC5, PC10); 5% and 10% RC (RC5, RC10); 10% of blended stabiliser with 20%RC/80%PC (RC2PC8) or 50%RC/50%PC (RC5PC5). The amount of stabiliser, 5 and 10 wt% of earth, covers the common range adopted in SCEB production [4,11,17,18]. Higher amounts of stabiliser lead to highly unsustainable solutions, with little improvement in performance [16,17].

The mixing water was defined based on w_opt_ (0) and adjusted with trial drop tests [58] and varied between 14.4% and 16.5%, depending on the type of binder. Due to the high water demand of RC, SCEBs with RC were produced with up to 10% more water than SCEBs with PC (Table 2).

**Figure 2:**
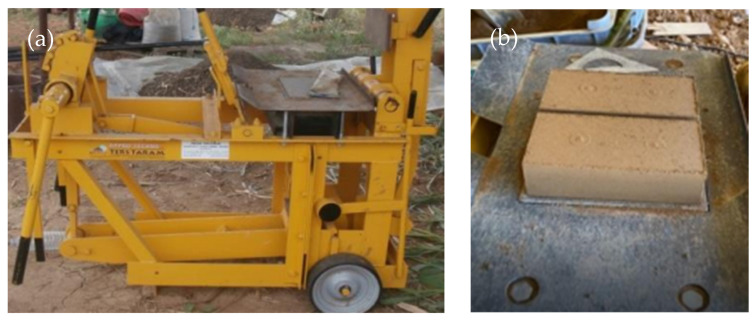
Compressed earthen block production: (**a**) manual press; (**b**) blocks just after being compressed.

All CEBs were produced with dimensions of 220 mm × 105 mm × 60 mm using a Terstaram manual press, (Appro Techno, Couvin, Belgium) of about 3.5 MPa maximum pressure capacity (Figure 2a). After demoulding (Figure 2b), CEBs stabilised with RC or PC were covered with a plastic film and sprayed with water twice a day for the first 7 days, while UCEBs were only covered with a plastic film without moistening. Afterwards, all CEBs were cured in a lab environment, at 55–70% relative humidity (RH), until testing.

### 2.4. Test Methods

The CEBs were characterised in terms of density, volume of voids, total porosity, compressive strength, water absorption by immersion, capillary water absorption, low-pressure water absorption, water permeability and water erosion resistance (spray test).

The fresh and hardened densities were determined according to EN 772-13 [59], considering 2 specimens per composition. From the values of fresh density and CEB composition (Table 2), it was possible to estimate the volume of voids left by the solids and mixing water after compaction (*V_V_*) and the mass proportion of each component. Total porosity (*P_T_*) was estimated from Equation (1), where *M_w_* and *ρ_w_* are the mass and density of water, respectively, and *M_b_*, *w_b_* and *α_H_* are the mass, the bound water after full hydration and the hydration degree of stabiliser, respectively. Basically, *P_T_* corresponds to the sum of *V_V_* and the volume of voids left by evaporated water and binder hydration. The *w_b_* was considered 0.23 for PC [60] and 0.22 for RC, because this binder is mainly composed of α’_H_-C_2_S, rather than C_3_S [32,50]. Moreover, a long-term hydration degree, α_H_, of 80% was assumed [30]. The solid volume of hydrated products was set equal to the sum of the volume of anhydrous binder and 0.746, relative to the volume of bound water [60]. This is an overestimate of *P_T_*, not considering shrinkage effects [60].

(1) $$P_{T}=Vv+\frac{\left[M_{w}-\alpha_{H}\times w_{b}\times 0.746\times M_{b}\right]}{\rho_{w}}\times 100$$

The compressive strength was tested at 28 days based on EN 772-1 [61] and Walker and Standards Australia [17]. Five blocks per composition were tested perpendicular to the bed face between two pieces of plywood and at a loading rate of about 4 kN/s. Tests were carried out on a Tonipact hydraulic press with 3000 kN capacity and a load cell with 200 kN capacity, due to the low strength of CEBs. In addition, water-saturated and oven-dried CEBs were also tested for the PC10 and RC10 compositions. This makes it possible to determine the loss of compressive strength after immersion in water, which is an indicator of the durability of SCEBs [62,63].

**Figure 3:**
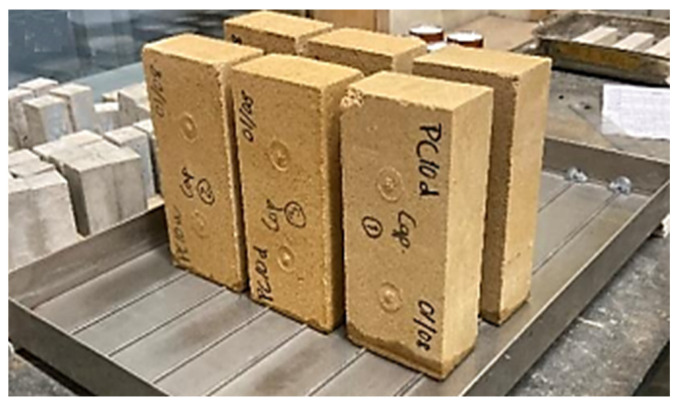
Capillary absorption test.

The water resistance of CEBs was accessed after 28 days. The immersion absorption was determined based on LNEC E394 [64] and NBR 8492 [65]. Two blocks per composition were immersed for 24 and 48 h, and then oven-dried at 100 ± 5 °C until constant mass. Water absorption corresponded to the water uptake by weight of the dry block. The capillary absorption was tested according to NTC 5324 [66] and EN 772-11 [67]. However, the blocks were tested in a vertical position to provide sufficient height, with the sides submerged up to 5 ± 1 mm (Figure 3). Before testing, three blocks per composition were oven-dried at 100 ± 5 °C until constant mass. Then, the blocks were placed in contact with water and weighed after 10 min, 20 min, 30 min, 60 min, 2 h, 6 h, 24 h, and 72 h.

The coefficient of capillary absorption, *C_b_*, was determined according to Equation (2), where *m_t_* (g) and *m_s_* (g) are the masses of the specimen at time *t* and when dried, respectively, *t* (min) is the time elapsed until measurement and *A* (m^2^) is the bottom area of the specimen in contact with water.

(2) $$C_{b}=\frac{\left(m_{t}-m_{s}\right)}{A\sqrt{t}}\times 100\;[g/{\mathrm{cm}}^{2}.\min^{0.5}]$$

**Figure 4:**
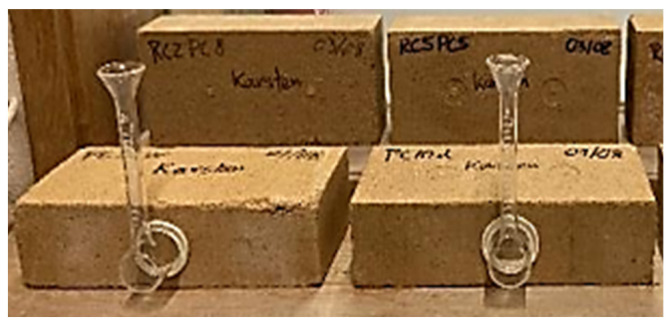
Low-pressure water absorption test.

The low-pressure water absorption was tested according to EN 16302 [68]. Karsten tubes were attached to the side of 3 blocks per composition and filled with 4 cm^3^ of water (Figure 4). The specimens were previously oven-dried at 100 ± 5 °C until constant mass. Then, the volume of water absorbed at different time intervals was recorded. The coefficient of absorption at low pressure, *C_abs,LP_*, corresponds to the slope of the linear interpolation between time and the ratio of the mass of water absorbed per area.

**Figure 5:**
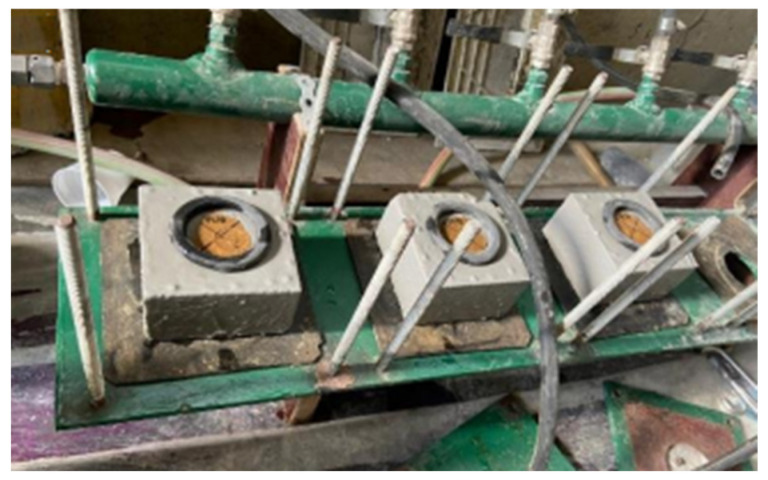
Water permeability test.

The water permeability was measured on three cut-half blocks of about 110 mm × 105 mm × 60 mm per composition, according to Bogas et al. [11]. After lab curing, the specimens were sealed with an epoxy-based paint, except for two 50 mm diameter areas on the top and bottom faces (Figure 5). Before testing, specimens were saturated in water to avoid capillary absorption phenomena. Then, the specimens were subjected to a constant pressure head of 100 kPa and, once the water flow had stabilised, the drained water was measured for 5 min. Rubber rings were fitted to the water inlets and outlets to prevent water leaks.

The permeability coefficient, *K_w_*, was calculated from Equation (3) according to Darcy’s law [69], which assumes laminar and permanent flow at a test temperature of 20 °C, where *Q* (m^3^/s) is the flow, *e* (m) is the specimen thickness, *S* (m^2^) is the available water penetration area and ∆*H* (kPa) is the pressure head.

(3) $$K_{w}=\frac{Q.e}{S.∆H}$$

**Figure 6:**
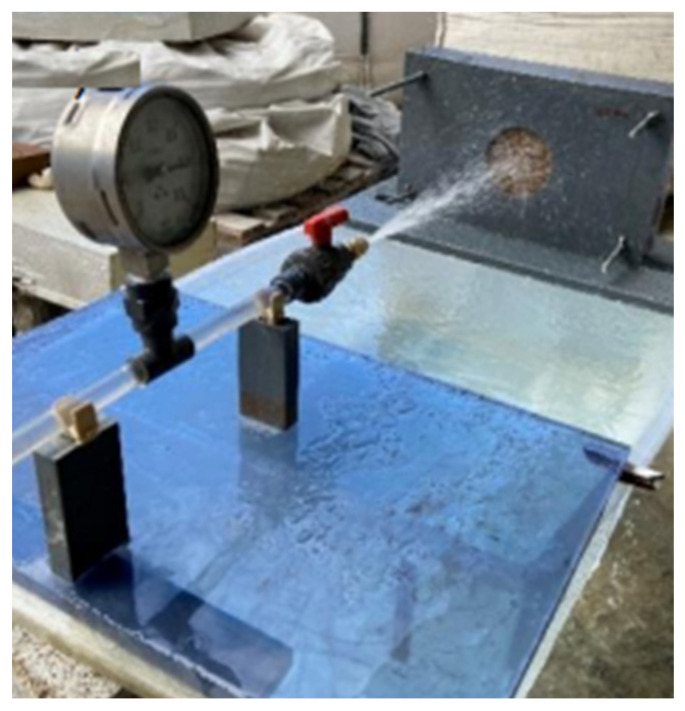
Water erosion resistance test.

The water erosion resistance was tested by means of spray tests according to NZS 4298 [58]. This test, which aims to simulate heavy rainfall, basically consisted of subjecting the blocks to a horizontal water jet with a pressure of 50 kPa for 1 h or until the erosion reached its full depth (Figure 6). To this end, a Fulljet GC-1550 nozzle was positioned 470 mm away from the block. Due to the relatively small size of specimens, the sprayed area was reduced from 140 mm to 100 mm in diameter. At the end, after sawing the specimen, the depth of erosion (*DE*), the erosion rate per hour (*DE*/hour) and the moisture penetration depth (*DP*) were measured. Two blocks were tested per composition. Tests were also carried out with a water pressure of 250 kPa to better distinguish the performance of the different types of CEB. Based on NZS 4298 [58] and considering a maximum CEB thickness of 60 mm, the following erosion indexes (EIs) are recommended [12]: *DE* < 10 (EI1); 10 ≤ *DE* < 25 (EI2); 25 ≤ *DE* < 45 (EI3); 45 ≤ *DE* < 60 (EI4); 60 ≤ *DE* (EI5).

In addition, thermogravimetric (TG) and XRD analyses were carried out on RC10 and PC10 SCEBs to better understand the influence of RC as a stabiliser. One sample of about 2 g was taken from the centre of half blocks per composition after 28 days, then ground and oven-dried at 40 °C for 48 h prior to testing. The TG analysis was performed on a *STERAM TGA92 Thermobalance*, under a nitrogen environment, where samples of about 60 mg were heated at 10 °C/min from ambient temperature up to 1000 °C. XRD was performed following the same procedure mentioned in Section 2.1.

## 3. Results and Discussion

### 3.1. Thermogravimetry and X-Ray Diffraction

**Figure 7:**
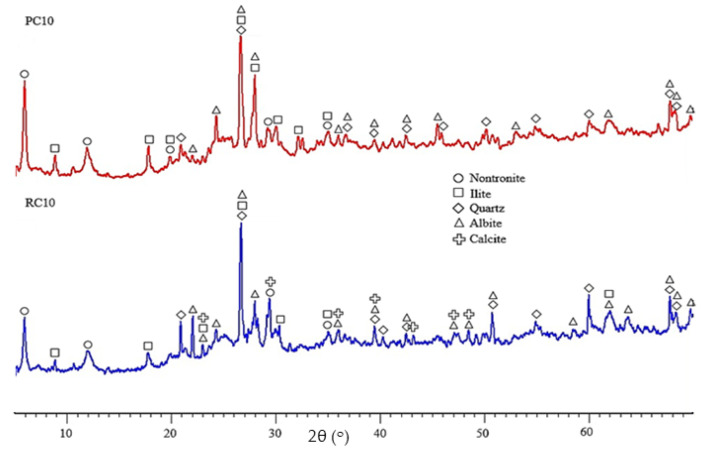
XRD analysis of PC10 and RC10.

Figure 7 shows the XRD diffractograms of PC10 and RC10. The minerals identified in both SCEBs were largely those found in the earth (Section 2.1), the most common being silicates, such as quartz (PDF 00-0-046-1045) and albite (PDF 00-041-1480). Clay minerals, namely illite (PDF 00-026-0911) and nontronite (PDF 00-029-1497), were also identified. The latter belongs to the group of smectite expansive clays, which are less stable than other clay minerals, such as kaolinite [70], due to their greater water adsorption and greater adverse effect on cement hydration. Therefore, the selected earth is less suitable for UCEB production [71]. Portlandite was not identified from XRD, due to its low amount, below the detection level [72]. The same happens with other less representative hydrated phases with low crystallinity, such as ettringite and AFm.

**Figure 8:**
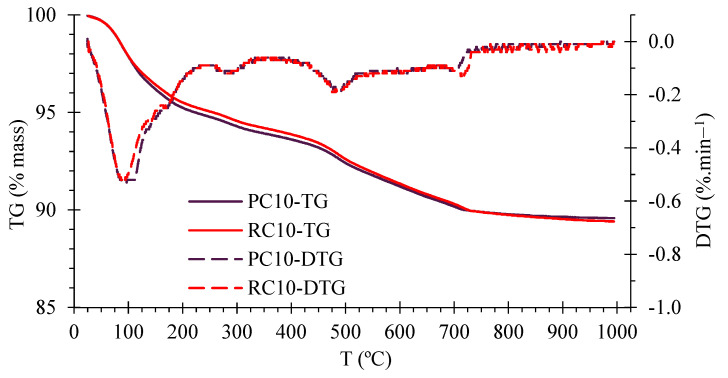
TG and DTG curves for PC10 and RC10.

Figure 8 shows the TG curve and its derivative (DTG) for both compositions. Weight losses are essentially related to the presence of cementitious materials and clay minerals. The TG and DTG curves were very similar in CEBs with PC or RC, indicating the development of the same type and amount of hydration phase. Three main regions of mass loss were identified, namely up to about 250 °C, between 420 °C and 540 °C and over 700 °C. In cementitious materials (PC and RC), these three phases are related to: elimination of free water and dehydration of hydration products (C-S-H, ettringite and Afm phases); dehydroxylation of calcium hydroxide; decarbonation of carbonated materials [73,74]. In addition, mass losses in the first two regions are also related to the elimination of hygroscopic water and dehydration of clay minerals [21], respectively. For clay minerals identified in XRD, mass loss peaks are reported to occur from 60–160 °C and 400–600 °C [75,76] in nontronite and at around 100 °C and 500–650 °C in illite.

As the CEBs were produced with 90% earth, it is difficult to interpret small differences related to the type of binder. Even so, the mass loss of PC10 was slightly higher than that of RC10, especially in the dehydration region, 100–420 °C (4.9% in PC10, 4.6% in RC10), suggesting that PC10 reached a slightly higher hydration degree. Indeed, the volume of anhydrous products available to hydrate tends to be higher in PC than in RC [32,36]. This is attributed to the higher volume of pre-carbonated compounds in RC10, as confirmed by the greater mass loss above 700 °C and the higher calcite content identified in the XRD (PDF 00-005-0586) (Figure 7).

Nevertheless, the effective rehydration of RC in SCEBs is demonstrated. The mass loss in the dehydroxylation phase was similar in PC10 and RC10 (0.6% from 420–540 °C), although it has been reported that calcium hydroxide tends to be higher in PC than in RC [30]. This is attributed to the overlapping mass losses of clay minerals. The low amount of calcium hydroxide (<2.5%) explains why it was not detected in XRD.

### 3.2. Density and Total Porosity

The fresh density, *ρ_f_*, immediately after CEB production varied between 1871 kg/m^3^ and 2026 kg/m^3^ (Table 3), within the range reported by other authors [11,77]. The highest compactness and fresh density were attained in UCEBs (Table 3), due to their lower water content (Table 2). On the other hand, RC SCEBs were produced with the highest water content (Table 2), due to the porous nature and high water demand of RC (Section 2.2). For this reason, the fresh density of RC SCEBs was about 4–6% lower than that of PC SCEBs. In fact, the high water demand of RC increased the w/b and made it more difficult to compact the RC SCEBs. In fact, RC-added CEBs presented higher *V_V_* (Table 3), confirming that they were less compacted than PC SCEBs, which affects their mechanical and durability properties. UCEBs had the lowest water content and *V_V_*. The total porosity, *P_T_*, of CEBs after long-term hydration is also presented in Table 3. The total porosity varied from 34.2–39.3%, with the lowest value obtained for the CEBs with 10% PC. The extra volume of hydration products compensated for the higher *V_V_* in PC SCEBs when compared to UCEBs. RC SCEBs had the highest *P_T_*, further confirming their lowest compactness.

**Figure 9:**
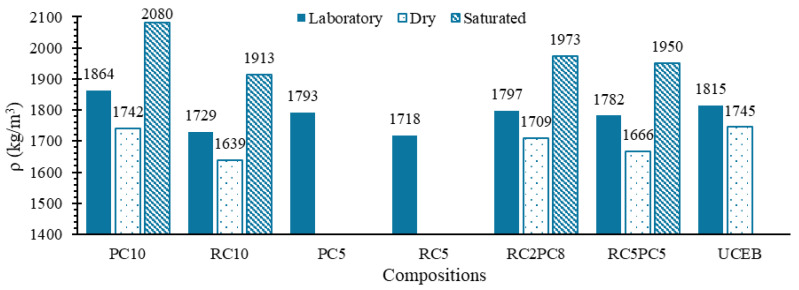
Hardened density (*ρ*) of CEBs for different water contents.

Figure 9 presents the density at 28 days, under dry, saturated and lab conditions. As expected, RC SCEBs had the lowest dry densities due their higher *P_T_* (Table 3). However, the difference between fresh density and dry density was slightly higher for SCEBs with PC (249 kg/m^3^) than with RC (232 kg/m^3^). This was not expected, because RC SCEBs were produced with higher water content and, as mentioned, the bound water tends to be slightly lower in RC than in PC [60]. The higher *P_T_* and lower volume of solids in RC SCEBs may explain the slightly lower amount of evaporated water compared to PC SCEBs. Regardless of this, it can be concluded that the amount of water evaporated was similar in SCEBs with RC or with PC, demonstrating the high rehydration capacity of RC. The saturated density of UCEBs is not presented in Figure 9 because, as expected, the blocks disintegrated after a few minutes of immersion in water. This also shows that RC was effective in stabilising the CEBs, maintaining the integrity of the immersed blocks. Compared to fresh density, the dry density of UCEBs was closer to that of SCEBs (Figure 9) because all water is evaporated, while in SCEBs part of the water reacts with the binder, increasing the density.

### 3.3. Compressive Strength

**Figure 10:**
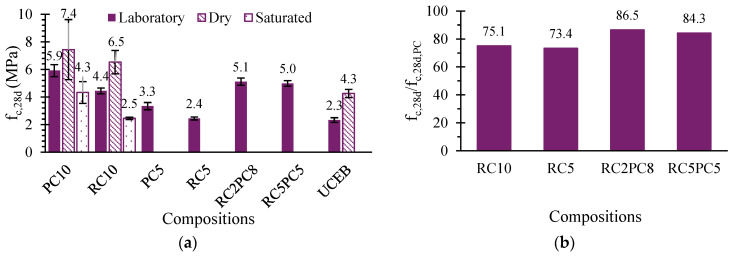
(**a**) Twenty-eight-day compressive strength (*f_c,28d_*) under dry, saturated and laboratory conditions; (**b**) relative twenty-eight-day compressive strength (*f_c,28d_*) in relation to reference PC SCEBs of equal binder content (*f_c,28d,PC_*).

The average 28-day compressive strength (*f_c_,_28d_*) after lab curing ranged between 2.3 and 5.9 MPa, depending on CEB composition (Figure 10a). SCEBs with 10% stabiliser were also tested in dry and saturated conditions because the mechanical strength of earthen materials depends greatly on their water content. UCEBs were not tested in saturated conditions since they fully disintegrated after immersion in water.

The compressive strength of CEBs depends on the geometry of the blocks, namely their height/width ratio (H/W). In fact, by varying this parameter between 0.5 and 5, the compressive strength can vary by more than double [78]. Therefore, correction factors depending on H/W are suggested to determine a reference unconfined compressive strength, *f_c,un_* [17,58]. For blocks tested with plywood boards to reduce the friction effect, Walker and Standards Australia [17] recommend the following correction factors to obtain *f_c,un_*: 1, 0.8, 0.7 and 0.5 for H/W of 5, 2, 1 and 0.4, respectively. In this study, the correction factor was 0.56 for H/W of 0.57. Therefore, the equivalent *f_c,un,28d_* varied from 1.3–3.3 MPa for lab curing (Table 3). For oven-dried specimens *f_c,un,28d_* was 2.4, 3.6 and 4.1 MPa for UCEB, RC10 and PC10, respectively (Figure 10a). This complies with the minimum 2 MPa recommended by Walker and Standards Australia [17].

The compressive strength increased with the stabiliser content and decreased with the progressive replacement of PC with RC (Figure 10a). SCEBs with 10% PC and 10% RC developed 2.5 and 1.9 times higher compressive strength than UCEBs, respectively. Therefore, RC has proven to be an effective alternative to PC, improving the bonding and cohesion between the earth particles.

**Figure 11:**
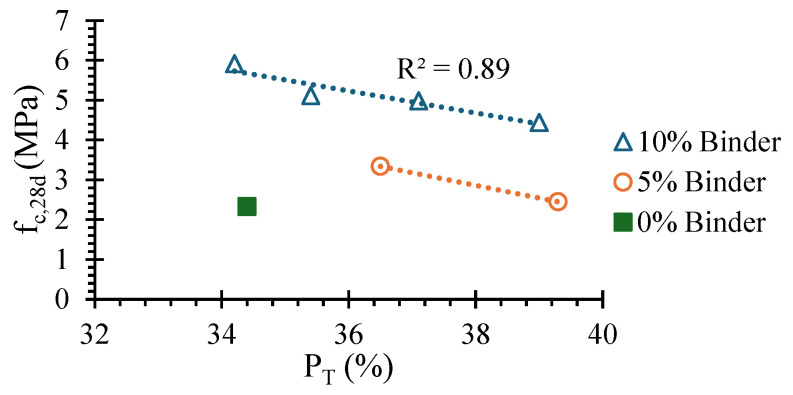
Correlation between SCEB average 28-day compressive strength (*f_c,28d_*) and total porosity (*P_T_*).

The compressive strength of RC SCEBs was around 73–75% of that obtained in SCEBs with the same amount of PC, being little affected by the binder content. As discussed, due to their greater water demand, RC SCEBs were produced with higher w/b and total porosity (Section 3.2). This can also be attributed to the formation of a smaller volume of long-term hydration products in RC than in PC [29,30]. This is in line with the reduction in compressive strength found in pastes with PC replaced with RC (Section 2.2). The reduction in compressive strength with RC content was almost linear. For 20% and 50% replacement of PC with RC, the reduction was only 13% and 16%, respectively (Figure 10b), related to a respective increase in *P_T_*. However, the correlation between *f_c,28d_* and *P_T_* is only high for the same amount of stabiliser, as the strength is also affected by the binding effect (Figure 11). Indeed, for SCEBs with PC or RC, the laboratory compressive strength increased by around 80% from 5% to 10% stabiliser content (Table 3). However, compared to UCEBs, the compressive strength increased 43% in PC5 but only 5% in RC5. This means that the binding effect provided by RC did not significantly compensate for the greater porosity of RC5 compared to PC5. Nevertheless, Figure 11 shows that, for the same *P_T_*, the compressive strength does not depend on the type of stabiliser, which means that RC had a similar hydration and binding capacity to PC. Moreover, for the same amount of PC and similar *P_T_*, the further addition of 5% RC (PC5 versus RC5PC5) led to 50% higher compressive strength. Therefore, the active contribution of RC to mechanical strength is demonstrated.

As expected, the compressive strength decreased in saturated SCEBs (Figure 10a), due to pore pressure effects and liquefaction of partly unstabilised clay [11,79]. SCEBs with PC or RC remained intact after saturation in water, which proves their stabilising capacity. However, they lost around 42% and 62% of their dry compressive strength, respectively. The presence of expansive clay minerals may have contributed to this poor behaviour in saturation conditions (Section 3.1). The reduction in compressive strength was greater in RC SCEBs than in PC SCEBs, increasing with the water content: 12%, 25% and 43% in dry, laboratory and saturated conditions, respectively. Thus, RC SCEBs were more affected by water content, which means that stabilisation with RC was less effective than with the same amount of PC. However, the reduction in dry and lab compressive strength was 43% and 61% higher in UCEBs than in RC SCEBs, respectively. Furthermore, unlike UCEBs, the RC SCEBs maintained their integrity under saturated conditions, which also confirms the stabilising action of RC.

As mentioned, the ratio between saturated and dry compressive strength (*f_c,s_/f_c,d_*) is a durability indicator [62,63]. In this case, the *f_c,s_/f_c,d_* was 0.58 and 0.38, for PC10 and RC10, respectively, which is higher than the minimum of 0.33 recommended for durable CEBs [11,62]. A slightly lower ratio of 0.53 was reported by Bahar et al. [47] in SCEBs with 10% PC. For the same compression pressure, Bogas et al. [11] found good water resistance in SCEBs produced with 4% PC and 4% air lime, where *f_c,s_/f_c,d_* was 0.33, lower than that obtained in RC10.

Bogas et al. [11] proposed the following reduction coefficients, *K_fc_*, for PC SCEBs, to take into account the variation in compressive strength with moisture content: 0.75 for SCEBs under 65% RH (*K_fc,65_*); 0.35 for SCEBs in contact with water (*K_fc,sat_*). In this study, the *K_fc,65_* and *K_fc,sat_* were 0.8 and 0.58 for PC SCEBs and 0.68 and 0.38 for RC SCEBs, respectively. Based on these results, the *K_fc_* recommended for RC SCEBs is suggested to be up to 0.1 lower than that of PC SCEBs.

### 3.4. Water Absorption by Immersion

**Figure 12:**
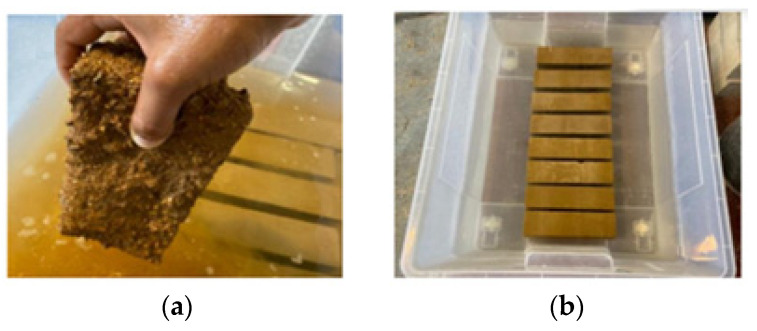
Blocks’ immersion absorption: (**a**) UCEB; (**b**) SCEBs with different types of stabilisers (RC, PC).

As mentioned in Section 3.3, unstabilised blocks gradually lost their cohesion after coming into contact with water (Figure 12a). Therefore, water absorption by immersion was only measured in SCEBs, which did not experience any apparent deterioration during the 48 h of immersion (Figure 12b).

**Figure 13:**
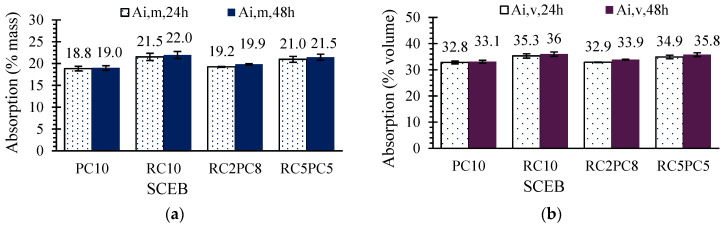
SCEB water absorption by immersion after 24 and 48 h: (**a**) in %mass (*A_i,m_*); (**b**) in %volume (*A_i,v_*).

**Figure 14:**
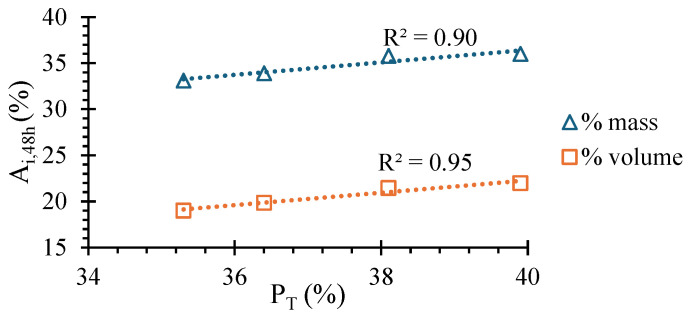
Correlation between SCEB average absorption by immersion after 48 h (*A_i,48h_*) and total porosity (*P_T_*).

Figure 13a,b show the average immersion absorption of SCEBs after 24 h and 48 h in terms of mass (*A_i,m_*) and volume percentage (*A_i,v_*), respectively. More than 95% of the 48 h absorption took place during the first 24 h. Absorption of SCEBs increased with RC content. *A_i,m_* and *A_i,v_* were up to 15% and 9% higher in RC10 than in PC10, respectively. The difference of *A_i,v_* was lower, because RC SCEBs had lower density than PC SCEBs. This parameter allows for a more accurate comparison between block compositions. The greater absorption of RC SCEBs is mainly attributed to their higher w/b and *P_T_* (14%). In fact, a high correlation was found between *P_T_* and *A_i,m_* or *A_i,v_* (Figure 14). Nevertheless, *A_i,v,48h_* increased by less than 3% for up to 50% RC content. *A_i,v_* corresponded to 92–97% of the total porosity estimated in Section 3.2. This indicates that over 92% of SCEB porosity was accessible to water within 48 h. The lowest water-accessible porosity ratio was obtained in RC10, which suggests a more refined porous structure (see Section 3.5).

A limit of 20% mass absorption at 24 h (*A_i,m,24h_*) is recommended by the NBR 8492 [65] for CEBs in a humid environment. In this study, only SCEBs with up to 20% substitution of PC by RC met this requirement. This may be because the SCEBs were produced with lower compactness than in other studies. Bogas et al. [11] reported 13.3% water absorption in 48 h for SCEBs with 8% PC and only 30% *P_T_*, which is in line with Figure 14. However, *A_i,m,24h_* was at the upper end of the usual absorption range (10–25%) according to Walker and Standards Australia [17].

### 3.5. Capillary Water Absorption

**Figure 15:**
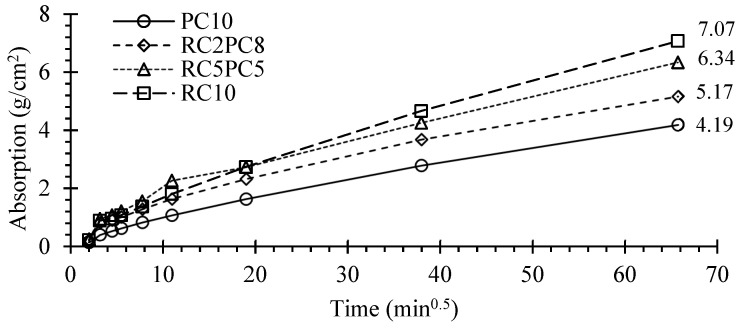
SCEB average capillary water absorption over time.

**Figure 16:**
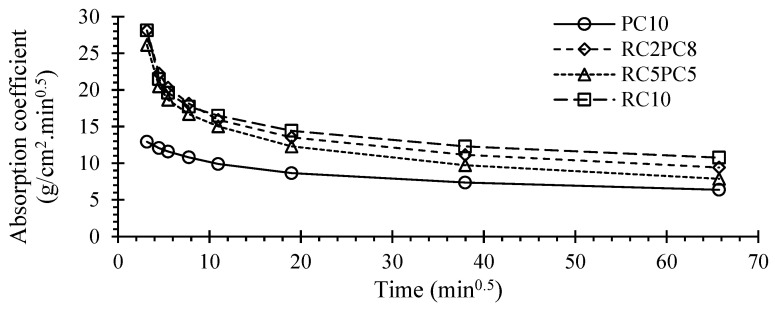
SCEB capillary absorption coefficient over time.

Figure 15 and Figure 16 show the average capillary absorption up to 72 h and the water absorption coefficient, *C_b_*, over time for SCEBs with 10% binder, respectively.

Despite the high coefficient of variation of this test, around 6–19%, the general trend was in line with previous tests (Section 3.4). The 72 h water absorption, *A_bs,72h_*, and the absorption rate increased with the replacement of PC with RC. The *A_bs,72h_* of RC10 was 69% higher than that of PC10 (Figure 15). In addition, the absorption coefficient after 30 min was 64–69% higher in RC10 (Figure 16). These differences were higher than those found in immersion absorption. On the one hand, the total porosity is higher in RC SCEBs, associated with higher *V_V_*, w/b and paste volume, which leads to a more open porous structure. On the other hand, for the same porosity, the microstructure tends to be more refined in pastes with RC than with PC [36]. Indeed, due to the porous nature of RC, RC pastes develop a dual microstructure made up of inner and outer particle porosity. As the porous RC retains part of the mixing water, the interparticle space becomes denser for the same total porosity [29]. As a result, the volume of narrow pores increases in RC pastes, increasing the capillary absorption action [50]. In addition, tests were carried out on oven-dried blocks, which can affect the microstructure, especially in less stable SCEBs with less PC. This is more relevant in the presence of expansive clays, with high shrinking and swelling properties. SCEBs with partial replacement of PC with RC showed intermediate absorptions compared to RC10 and PC10. However, the absorption coefficient at 10 min, *C_b,10 min_*_,_ was little affected in SCEBs with more than 20% RC. Therefore, after pre-drying, the incorporation of RC increased the pore connectivity, leading to faster absorption. Indeed, converting *A_bs,72h_* into a percentage of volume, the capillary absorption involved 56% and 83% of the total porosity in PC10 and RC10, respectively.

According to NTC 5324 [66], CEBs can be classified based on their *C_b,10min_*: I—*C_b,10min_* ≤ 20 g/cm^2^.min^0.5^—“very low absorption”; II—*C_b,10min_* ≤ 40 g/cm^2^.min^0.5^—“low absorption”. Even tested perpendicular to the compaction direction, SCEBs with 10% PC fell into class I (*C_b,10min_* = 12.9 g/cm^2^.min^0.5^) and SCEBs with 10% RC into class II (*C_b,10min_* = 28.1 g/cm^2^.min^0.5^). Despite its lower performance than PC, the incorporation of RC allowed the production of low-absorption SCEBs.

### 3.6. Low-Pressure Water Absorption by Karsten Tubes

**Figure 17:**
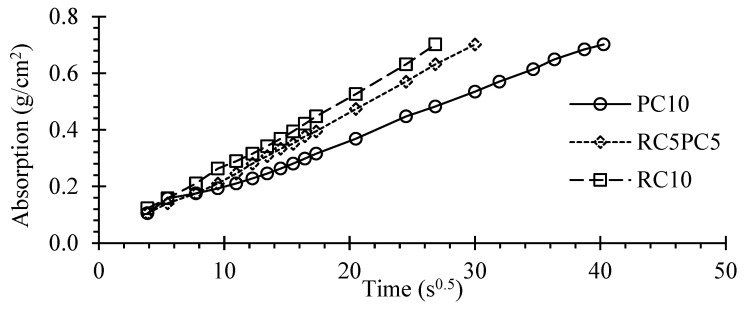
SCEB water absorption at low pressure over time.

The low-pressure water absorption over time is presented in Figure 17. This test is affected by surface absorption and permeability phenomena. As found in Section 3.5, PC10 had the lowest absorption rate over time. Compared to PC10, the time to absorb 4 cm^3^ of water by a defined area (0.7 g/cm^2^) was 56% and 48% lower in RC10 and RC5PC5, respectively (Figure 17). Moreover, the absorption coefficient at 5 min, *C_abs,LP,5min_*, was 50% higher in RC10 (0.012 kg.m^−2^.s^−1^) than in PC10 (0.008 kg.m^−2^.s^−1^). Thus, the higher absorption rate of SCEBs with RC, especially after pre-drying, is confirmed. As discussed in Section 3.5, the higher P_T_ increased the pore connectivity, and the greater volume of narrow pores increased the absorption properties.

### 3.7. Water Permeability

**Figure 18:**
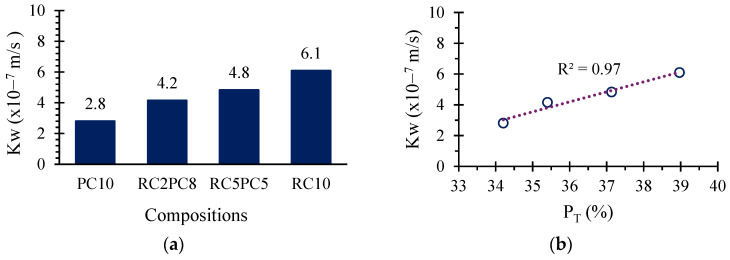
SCEB water permeability coefficient (KW) (**a**) and versus total porosity (*P_T_*) (**b**).

The average permeability coefficient (*K_W_*) varied between 2.8 × 10^−7^ m/s, for SCEBs with 10% PC, and 6.1 × 10^−7^ m/s, for SCEBs with 10% RC (Figure 18a). Values in the same range were reported in previous studies [11,12] involving SCEBs with 8% PC (2.6–3.2 × 10^−7^ m/s). Bahar et al. [47] obtained a lower *K_W_* in stabilised compacted earthen cylinders with 10% PC (0.8 × 10^−7^ m/s), which is explained by the higher compaction pressure used in their production. Note that medium- to low-quality concrete has *K_W_* values of about 10^−16^–10^−10^ m/s [80], 3–9 orders of magnitude lower than SCEBs. Therefore, water permeability is much more important in CEBs than in concrete.

The permeability increased with the progressive replacement of PC with RC (Figure 18a), confirming the general trend observed in the other tests. *K_W_* was about 2 times higher in SCEBs with 10% RC than with 10% PC (Figure 18a). This coefficient correlated well with total porosity (Figure 18b), demonstrating the importance of this parameter in the durability of SCEBs. However, the difference in permeability was much greater than that found in *P_T_* and in water absorption. This is because permeability is more affected by pore connectivity and water absorption than by total porosity. In fact, a small increase of 14% in total porosity (Section 3.2) corresponded to a significant increase in permeability when PC was replaced with RC (Figure 18a). According to Powers [81], increasing the porosity of cement pastes by 14% can more than double the water permeability. This confirms that pore connectivity increased significantly with RC incorporation, mainly due to the production of less compacted blocks, with higher *P_T_*. Therefore, future research should focus on optimising the mix design and compaction by compression of RC SCEBs, since RC showed a high hydration and binding capacity (Section 3.3).

However, increasing permeability to intermediate levels compared to UCEBs and PC SCEBs can be beneficial in terms of hygrothermal inertia, drying capacity and water vapour permeability [82], although these properties were not assessed in this study.

### 3.8. Resistance to Water Erosion by Spray Test

**Figure 19:**
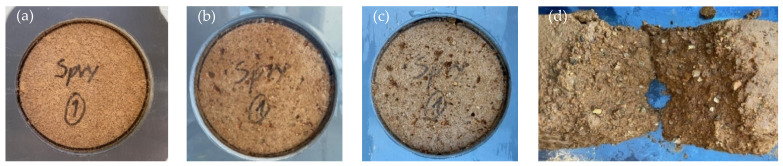
CEB before and after the spray test: (**a**) PC10 before testing; (**b**,**c**) minor surface erosion of PC10 and RC10 after 1 h testing at 2.5 bar, respectively; (**d**) fully eroded unstabilised CEB after 7 min at 0.5 bar.

Regardless of the type of binder, stabilised blocks had very good durability when subjected to the spray test (Table 4). According to NZS 4298 [58], all SCEBs showed no signs of erosion when exposed to 0.5 bar pressure, which simulates heavy rain impact. All the blocks tested had erosion rates of less than 1 mm/h, falling within the best durability class IE1 (Section 2.4). Exelbirt [83] also reported erosion rates below 1 mm/h for SCEBs with 7% cement, but 25 mm/h for blocks with 5% cement and 7% lime. This shows the good efficiency of RC used in this study. Even when the water jet pressure was increased 5 times to 2.5 bar, erosion was not significant after 1 h of testing (Table 4). In this situation, the SCEBs suffered only minor surface erosion, losing some particles up to circa 2 mm in size (Figure 19). The level of surface erosion appeared to have been slightly higher in RC10 than in PC10, although the differences were not significant.

The depth of moisture penetration (*DP*) was also measured after the test by sectioning the blocks perpendicular to the exposed face (Table 4). The *DP* was little affected by the type of stabiliser (Table 4). Similar values of *DP* (40 mm) were reported by Bogas et al. [11] in SCEBs with 8% PC. In addition, the RC5PC5 behaved like SCEBs with 10% PC (Table 4), also showing the effective participation of RC. Note that, according to the literature, these tests simulate more severe conditions than those commonly found in real exposure environments [83,84]. This highlights the good water erosion resistance of SCEBs with RC when compared to PC-added SCEBs.

On the other hand, unstabilised blocks were completely eroded after just 7 min of testing at the lowest pressure of 0.5 bar (Table 4, Figure 19d). These blocks were very susceptible to water erosion, disintegrating quickly on contact with water. Other authors have documented similar behaviour in UCEBs [11,12,83,85]. For this reason, UCEBs are not recommended for unprotected outdoor applications. This emphasises the importance of stabilisation and the good efficiency of recycled cement for outdoor unrendered masonries.

The results of this study are very promising, especially given the high potential for reducing the environmental impact of stabilised earth compressed blocks. Considering the SCEB compositions in Table 2, and assuming that CO_2_ emissions in RC production are 70% lower than in PC production [33], SCEBs with 20%, 50% and 100% RC can potentially save up to 16%, 38% and 72% of CO_2_, respectively, compared to reference PC SCEBs. Moreover, natural resources are saved and construction waste is reused. Therefore, RC proved to be a very promising alternative to PC for the production of SCEBs.

## 4. Conclusions

Although RC has been proven to efficiently improve the mechanical properties of unstabilised CEB, the durability behaviour was yet to be evaluated. This study analysed the effect of a new low-carbon alternative for PC, namely recycled cement (RC), on the water resistance of stabilised compressed earth blocks (SCEBs). SCEBs with RC were produced with higher w/b and lower compactness by compression, due to their greater water demand. As a result, total porosity, *P_T_*, and pore connectivity increased, affecting mechanical strength and durability.

The durability indicator, *f_c,s_/f_c,d_*, was lower in RC SCEBs than in PC SCEBs, indicating that stabilisation with RC was less effective than with the same amount of PC. Even so, this ratio was over the minimum recommended in the literature for durable SCEBs.

Water absorption and water permeability increased when PC was replaced by RC. Although long-term immersion absorption increased by less than 10%, the capillary absorption rate and the water permeability coefficient increased by up to 70% and 100%, respectively. The higher *P_T_* and greater volume of narrow pores in SCEBs with RC increased their permeability and absorption rate, especially after pre-drying.

However, RC showed high rehydration capacity and binding efficiency similar to that of PC. For the same porosity, the compressive strength was little affected by the type of stabiliser. Moreover, the incorporation of RC doubled the compressive strength of unstabilised CEBs and made them water resistant. Although CEBs were produced with earth composed of expansive clay minerals, RC was effective for their stabilisation, maintaining their integrity even after 48 h of water immersion and a severe water erosion test. Unlike unstabilised CEBs, RC SCEBs complied with normative requirements and were shown to be viable even for unprotected outdoor applications.

Even tested in the dry state and perpendicular to the direction of compaction, RC SCEBs fell into the “low-absorption” class. In addition, the RC SCEBs showed high resistance to water erosion, even when the water pressure was increased by 5 times, simulating much harsher conditions than those found in common real environments. The erosion rate and the depth of moisture penetration were little affected by the type of stabiliser used.

In conclusion, sustainable and water-resistant SCEBs have been produced with a local earth and up to 100% low-carbon recycled stabiliser, reducing carbon emissions by more than 70% compared to PC SCEBs. Recycled cement has proved to be a very promising alternative to raw cement, increasing the eco-efficiency of stabilised compressed earth blocks without significantly compromising their water resistance. Future studies should explore more optimising compositions that can mitigate the high water demand of RC and consequent production of CEBs with lower compactness.

## Figures and Tables

**Table 1:** Properties of Portland (PC) and recycled (RC) cements.

Parameters		Standard	PC	RC
Density (g/cm^3^)		^a^	3.07 ± 0.3	3.00
Compressive strength of reference mortar (MPa)	2 days	EN 196-1 [52]	16.80 ± 1.2	-
	28 days		57.00 ± 3.8	-
SiO_2_ + Al_2_O_3_ + Fe_2_O_3_ (%)		EN 196-2 [51]	19.64 + 5.34 + 3.05	19.14 + 5.13 + 3.00
CaO + MgO (%)			62.80 + 1.80	60.79 + 1.77
Free CaO		EN 451-1 [53]	0.70	13.94
Water demand (w/b)		EN 196-3 [54]	0.31	0.73
Setting time (min)	Initial	EN 196-3 [54]	170	290
	Final		280	385

^a^ Through measurement by helium pycnometer.

**Table 2:** Unstabilised and stabilised compressed earth block composition.

Mixture	Soil ^a^ (%)	PC ^b^ (%)	RC ^b^ (%)	Water ^b^ (%)	w/b ^c^
PC10	90	10	-	15.0	1.45
RC10	90	-	10	16.5	1.60
PC5	95	5	-	15.2	2.93
RC5	95	-	5	16.2	3.13
RC2PC8	90	2	8	15.0	1.45
RC5PC5	90	5	5	15.5	1.50
UCEB	100	-	-	14.4	-

^a^ Earth with 4% humidity; ^b^ by weight of solids (earth + stabiliser); ^c^ total water/binder, including water absorbed by the earth.

**Table 3:** Composition, average fresh and lab density, void volume, long-term total porosity and average values and coefficients of variation of compressive strength.

Mixture	M_PC_ (kg/m^3^)	M_RC_ (kg/m^3^)	M_S_ (kg/m^3^)	ρ_f_ (kg/m^3^)	ρ_lab,28d_ (kg/m^3^)	V_V_ (%)	*P_T_* (%)	*f_c,28d_* (MPa)	CV_fc,28d_ (%)	*f_c,un,28d_* (MPa)
PC10	179	-	1552	1991	1864	10.7	34.2	5.92	4.5	3.30
RC10	-	166	1439	1871	1729	14.6	39.0	4.44	6	2.47
PC5	88	-	1605	1950	1793	12.0	36.5	3.34	8.8	1.86
RC5	-	84	1532	1879	1718	14.2	39.3	2.45	4.8	1.36
RC2PC8	141	35	1523	1954	1797	12.4	35.4	5.12	5.6	2.85
RC5PC5	86	86	1482	1909	1782	13.9	37.1	4.99	2.3	2.78
UCEB	-	-	1771	2026	1815	8.9	34.4	2.33	8.3	1.30

ρ_f_—average fresh density; ρ_lab_—average lab density; *f_c,28d_*—average 28-day compressive strength; *f_c,un,28d_*—unconfined *f_c,28d_*; M_PC_—cement mass; M_RC_—recycled cement mass; M_S_—mass of dry earth; *P_T_*—estimated long-term total porosity; V_V_—void volume.

**Table 4:** CEB erosion Depth (DE), moisture penetration Depth (DP) and erosion rate (DE/hour) by spray test.

Mixture	Pressure (bar)	Test Time (min)	DE (mm)	Erosion Rate (mm/hour)	DP (mm)
PC10	2.5	60	-	<1	38
RC5PC5	2.5	60	-	<1	36
RC10	2.5	60	-	<1	39
UCEB	0.5	7	60	514	Fully eroded

## Data Availability

The original contributions presented in the study are included in the article, further inquiries can be directed to the corresponding author/s.
